# Role and Mechanism of cGAS-STING Pathway in Cardiovascular System

**DOI:** 10.31083/j.rcm2504135

**Published:** 2024-04-07

**Authors:** Xianqiang Yu, Silin Pan

**Affiliations:** ^1^Heart Center, Women and Children's Hospital Affiliated to Qingdao University, 266034 Qingdao, Shandong, China

**Keywords:** cardiovascular disease, STING, cGAS, inflammation, endothelial cell

## Abstract

The cyclic guanosine monophosphate-adenosine monophosphate (GMP-AMP) synthase (cGAS)-stimulator of interferon genes (STING) pathway is a part of the innate immune system that plays a role in the cardiovascular system. It acts as a surveillance system, detecting and responding to cytosolic DNA, viral DNA, and other intracellular DNA species. Activation of the cGAS-STING pathway leads to the production of inflammatory cytokines and type I interferons, which are involved in the immune response. In the cardiovascular system, the cGAS-STING pathway has been implicated in various physiological and pathological processes. It contributes to vascular inflammation, atherosclerosis, endothelial dysfunction and cardiac remodeling and heart failure. In this review, we will elaborate on the research progress of the role of cGAS-STING in cardiovascular system.

## 1. Introduction

Cardiovascular disease (CVD) refers to a class of disorders that involve the heart and blood vessels, impairing their structure or function [1, 2, 3, 4]. CVDs can lead to various complications, including angina, heart failure, stroke, and peripheral artery disease. These conditions can cause disability, reduced quality of life, and functional limitations [5, 6]. Therefore, CVDs are a leading cause of death globally and impose a significant burden on healthcare systems. Exploring the molecular mechanism of cardiovascular disease has always been the focus of research. However, CVDs involve complex molecular mechanisms that contribute to their development and progression.

Inflammation plays a complex role in CVD. It can both contribute to the development and progression of CVD and be a consequence of these conditions. The relationship between inflammation and CVD is multifaceted. Therefore, inflammation is an important factor in cardiovascular disease, and its management is an area of ongoing research [7, 8]. Reducing chronic inflammation through lifestyle changes and, in some cases, medication may help alleviate symptoms and reduce the risk of cardiovascular events. However, individual approaches should be tailored to a person’s specific cardiovascular risk factors and overall health.

**Fig. 1.**
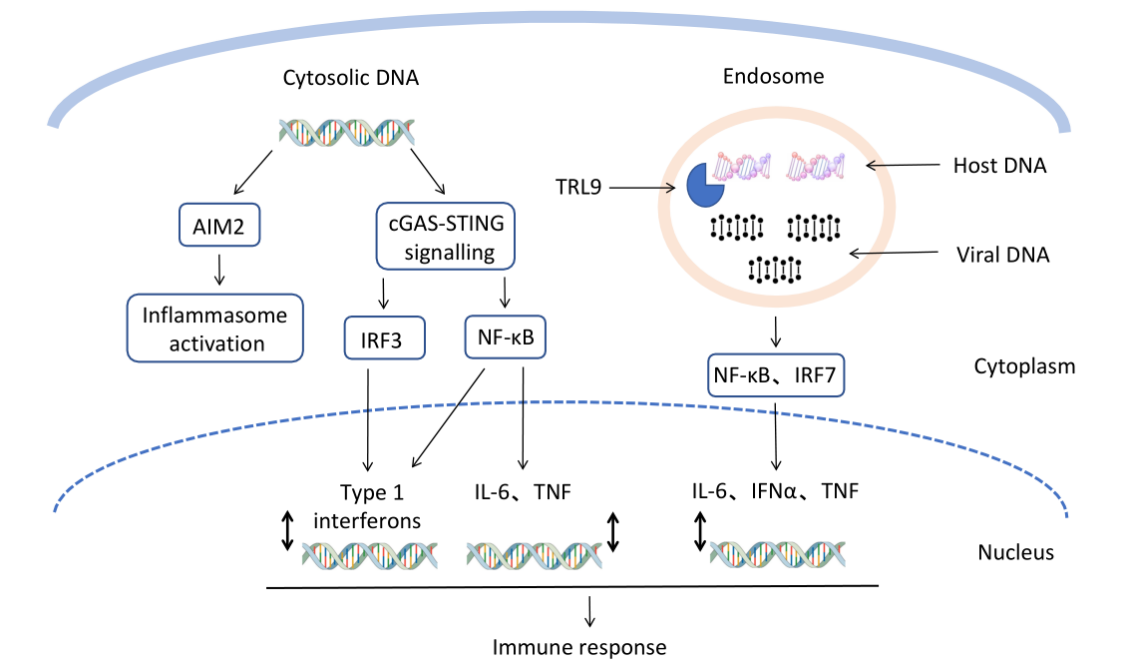
**Schematic diagram of cGAS-STING pathway**. cGAS, cyclic GMP-AMP synthase; STING, stimulator of interferon genes; IRF, interferon regulatory factor; NF-κB, nuclear factor κB; TRL9, toll-like receptor 9; IL-6, interleukin-6; IFNα, interferon α; TNF, tumor necrosis factor; AIM2, absent in melanoma 2.

The cGAS-STING pathway, consisting of cyclic guanosine monophosphate-adenosine monophosphate (GMP-AMP) synthase (cGAS) and stimulator of interferon genes (STING), is primarily known for its role in innate immunity and the detection of cytosolic DNA [9, 10]. In 2008, a study published in Nature identified cGAS as a potential cytosolic DNA sensor. Researchers showed that cGAS was capable of detecting foreign DNA in the cytoplasm and triggering an immune response [9]. A significant breakthrough came in 2012 when a study published in Nature reported the discovery of cyclic GMP-AMP (cGAMP) as the second messenger produced by cGAS upon binding to DNA. cGAMP was shown to be essential for the activation of downstream signaling. During the same period, STING was identified as an essential component of the pathway. STING acts as a signaling adaptor that interacts with cGAMP and activates downstream signaling events [9]. The cGAS-STING pathway in the cardiovascular system can be activated by a broad range of stimuli and situations, including organelle damage, DNA damage, infections, and various cellular stresses. The cGAS-STING pathway acts as a key component of the innate immune system, sensing the presence of cytosolic DNA derived from microbial infections or cellular damage. Upon binding to dsDNA, cGAS catalyzes the production of cGAMP, a second messenger molecule [11]. The produced cGAMP binds to and activates STING, leading to its oligomerization and activation. Activated STING recruits downstream signaling molecules, including TANK-binding kinase 1 (TBK1) and interferon regulatory factor 3 (IRF3) (Fig. 1). TBK1 phosphorylates IRF3, which results in its nuclear translocation and activation. Activated IRF3 induces the transcription of genes involved in immune responses, including the production of type I interferons (such as interferon-beta) and other pro-inflammatory cytokines [12, 13]. Type I interferons play a crucial role in antiviral defense, promoting an antiviral state in infected cells and activating immune responses. The cGAS-STING pathway also activates immune cells, such as natural killer (NK) cells, macrophages, and dendritic cells, contributing to the clearance of viral infections [14]. However, emerging evidence suggests that this pathway also plays important roles in the cardiovascular system. In this review, we will elaborate on the research progress of the role of cGAS-STING pathway in cardiovascular system.

## 2. Mechanism

### 2.1 Vascular Inflammation

The cGAS-STING pathway has been implicated in vascular inflammation, playing a role in the pathogenesis of various cardiovascular diseases. Activation of the cGAS-STING pathway in vascular cells, including endothelial cells and smooth muscle cells, results in the production of pro-inflammatory cytokines [15, 16]. This includes the production of interleukin-6 (IL-6), tumor necrosis factor-alpha (TNF-alpha), and interferons. These cytokines contribute to the inflammatory response in the vascular wall, promoting leukocyte recruitment, activation of immune cells, and amplifying the inflammatory cascade. The cGAS-STING pathway activation in vascular cells can lead to the release of chemotactic factors and damage-associated molecular patterns (DAMPs), which can activate immune cells, such as macrophages and dendritic cells [17]. Activated immune cells infiltrate the vascular wall, further spreading inflammation and contributing to the progression of vascular diseases. Activation of the cGAS-STING pathway can induce oxidative stress in vascular cells [18, 19]. Increased production of reactive oxygen species (ROS) and impaired antioxidant defense mechanisms lead to oxidative damage to the vascular wall. Therefore, the excessive production of ROS can lead to damage of cellular components, including lipids, proteins, and DNA, as well as oxidative modification of low-density lipoprotein (LDL) cholesterol, inflammation, endothelial dysfunction, and tissue injury [18]. As a result, ROS contribute to the pathogenesis and progression of various cardiovascular diseases, including atherosclerosis, hypertension, heart failure, and ischemic heart diseases. Therefore, controlling ROS and oxidative stress is an important therapeutic target in the management of cardiovascular diseases. Antioxidant strategies and lifestyle modifications may help mitigate the detrimental effects of ROS in the cardiovascular system. ROS production in cardiovascular disease can result from a combination of mechanisms, including mitochondrial dysfunction, activation of NADPH oxidase (NOX) enzymes, and a loss of antioxidant function. These mechanisms often work in concert to create an environment of oxidative stress in the cardiovascular system [19]. It’s important to note that these mechanisms are interconnected and can amplify one another. Mitochondrial dysfunction and NOX activation can lead to a chain reaction of ROS production, further exacerbating oxidative stress. Similarly, a loss of antioxidant function can leave the cardiovascular system vulnerable to ROS damage. Overall, understanding the complex interplay of these mechanisms is crucial for developing targeted therapies to mitigate oxidative stress and its role in cardiovascular diseases.

The dysregulation of the cGAS-STING pathway in vascular inflammation is implicated in various cardiovascular diseases, including atherosclerosis, hypertension, and vascular injury. Targeting this pathway may hold therapeutic potential for mitigating vascular inflammation and preventing or treating related cardiovascular pathologies. However, further research is needed to fully understand the complex interplay of the cGAS-STING pathway with other inflammatory signaling pathways in vascular inflammation and to develop effective therapeutic strategies.

### 2.2 Atherosclerosis

The cGAS-STING pathway has emerged as a significant player in the development and progression of atherosclerosis, a chronic inflammatory disease characterized by the accumulation of plaque in the arterial wall [20]. The cGAS-STING pathway is activated in various cell types within the atherosclerotic plaque, including endothelial cells, vascular smooth muscle cells, and immune cells [21, 22]. It detects the presence of cytosolic DNA derived from damaged cells, oxidized low-density lipoprotein (LDL), or microbial pathogens in the vessel wall. Activation of the cGAS-STING pathway in endothelial cells leads to the upregulation of adhesion molecules, such as vascular cell adhesion molecule-1 (VCAM-1) and intercellular adhesion molecule-1 (ICAM-1), promoting leukocyte adhesion and infiltration into the arterial wall [23]. This contributes to the initiation of early atherosclerotic lesions. Activation of the cGAS-STING pathway in macrophages can enhance lipid uptake and foam cell formation, a hallmark of atherosclerosis. Foam cells are lipid-laden macrophages that contribute to the progression of the plaque. The cGAS-STING pathway activation triggers the production of type I interferons, particularly interferon-beta (IFN-beta), within the plaque [24, 25]. Type I interferons have been shown to promote plaque development and instability by modulating the immune response and promoting the expression of matrix metalloproteinases (MMPs), which can weaken the fibrous cap of the plaque. In addition, the cGAS-STING pathway activation can induce oxidative stress within the plaque, leading to the production of ROS as aforementioned [23]. Oxidative stress further promotes inflammation, endothelial dysfunction, and lipid oxidation, contributing to plaque progression.

The dysregulation of the cGAS-STING pathway in atherosclerosis suggests its involvement in multiple stages of plaque development and progression. Targeting this pathway may hold therapeutic potential for modulating inflammation, reducing plaque burden, and stabilizing vulnerable plaques.

### 2.3 Endothelial Dysfunction

The cGAS-STING pathway has been implicated in the development of endothelial dysfunction, a key pathological feature of various cardiovascular diseases. Activation of the cGAS-STING pathway in endothelial cells can trigger an inflammatory response [26]. This includes the upregulation of adhesion molecules, such as vascular cell adhesion molecule-1 (VCAM-1) and intercellular adhesion molecule-1 (ICAM-1), on the endothelial cell surface [27, 28]. Increased expression of these molecules promotes the adhesion and infiltration of immune cells, such as monocytes and lymphocytes, into the vessel wall. Activation of the cGAS-STING pathway in endothelial cells can induce oxidative stress [29]. This results in the generation of ROS and a disruption of the balance between oxidants and antioxidants. Excessive ROS production contributes to endothelial dysfunction by impairing endothelial nitric oxide (NO) bioavailability, a critical regulator of vascular tone and function [30]. NO is produced by endothelial nitric oxide synthase (eNOS) and acts as a vasodilator, anti-inflammatory, and anti-thrombotic molecule. Activation of the cGAS-STING pathway can lead to reduced eNOS activity and decreased NO production, contributing to endothelial dysfunction and impaired vasodilation.

The dysregulation of the cGAS-STING pathway in endothelial cells can contribute to endothelial dysfunction, a crucial step in the development and progression of cardiovascular diseases such as atherosclerosis, hypertension, and vascular inflammation. Targeting this pathway may hold therapeutic potential for preserving endothelial function and preventing the progression of cardiovascular pathologies.

### 2.4 Cardiac Remodeling and Heart Failure

The cGAS-STING pathway has been recognized as a significant contributor to cardiac remodeling and the development of heart failure, a complex and progressive cardiovascular disorder. Activation of the cGAS-STING pathway in cardiac cells, such as cardiomyocytes and fibroblasts, leads to the production of pro-inflammatory cytokines, including interleukin-1 beta (IL-1β), TNF-alpha, and interferons [31, 32, 33]. This initiates an inflammatory response in the heart, promoting infiltration of immune cells and the activation of cardiac fibroblasts. Persistent activation of cardiac fibroblasts results in excessive collagen deposition, leading to cardiac fibrosis, which contributes to impaired cardiac function and remodeling. At the same time, activation of the cGAS-STING pathway in cardiomyocytes can result in cellular dysfunction [34]. Increased production of pro-inflammatory cytokines, oxidative stress, and mitochondrial dysfunction can lead to cardiomyocyte apoptosis, impaired contractility, and compromised cardiac function [32]. Conversely, activation of the cGAS-STING pathway can also promote cell survival pathways, such as autophagy, as a protective response. The balance between cell death and survival mechanisms in response to cGAS-STING pathway activation influences cardiac remodeling and heart failure progression. In addition, activation of the cGAS-STING pathway in cardiac fibroblasts promotes their differentiation into myofibroblasts, which are responsible for excessive collagen production and deposition in the cardiac tissue [32, 35, 36]. Myocardial fibrosis disrupts the normal architecture of the heart and impairs its mechanical function.

The dysregulation of the cGAS-STING pathway in the heart contributes to pathological cardiac remodeling, characterized by inflammation, fibrosis, cardiomyocyte dysfunction, and impaired contractility, ultimately leading to heart failure. Understanding the intricate mechanisms underlying the cGAS-STING pathway in cardiac remodeling may pave the way for novel therapeutic strategies targeting this pathway to alleviate heart failure progression.

## 3. Conclusions

The cGAS-STING pathway is an innate immune signaling pathway that plays a role in the cardiovascular system. It functions as a surveillance system to detect cytosolic DNA, viral DNA, and other intracellular DNA species. When activated, the cGAS enzyme recognizes and binds to these DNA molecules, leading to the production of cGAMP. cGAMP then binds to the STING protein, activating downstream signaling pathways. In the cardiovascular system, the cGAS-STING pathway has been implicated in various physiological and pathological processes, including vascular inflammation, atherosclerosis, endothelial dysfunction and cardiac remodeling and heart failure. Understanding the role of the cGAS-STING pathway in the cardiovascular system provides insights into the pathogenesis of cardiovascular diseases and may offer potential therapeutic targets for intervention. However, further research is needed to fully elucidate the intricate mechanisms and clinical implications of this pathway in cardiovascular health and disease.
